# It Takes a Community to Raise the Prevalence of a Zoonotic Pathogen

**DOI:** 10.1155/2011/741406

**Published:** 2011-11-21

**Authors:** Dustin Brisson, Catherine Brinkley, Parris T. Humphrey, Brian D. Kemps, Richard S. Ostfeld

**Affiliations:** ^1^Department of Biology, University of Pennsylvania, Leidy Laboratories 209, 433 South University Avenue, Philadelphia, PA 19104-6018, USA; ^2^Department of Ecology and Evolution, University of Arizona, BioSciences West room 310, 1041 E. Lowell St., Tucson, AZ, USA; ^3^Cary Institute of Ecosystem Studies, Box AB, Millbrook, NY 12545, USA

## Abstract

By definition, zoonotic pathogens are not strict host-species specialists in that they infect humans and at least one nonhuman reservoir species. The majority of zoonotic pathogens infect and are amplified by multiple vertebrate species in nature, each of which has a quantitatively different impact on the distribution and abundance of the pathogen and thus on disease risk. Unfortunately, when new zoonotic pathogens emerge, the dominant response by public health scientists is to search for a few, or even the single, most important reservoirs and to ignore other species that might strongly influence transmission. This focus on the single “primary” reservoir host species can delay biological understanding, and potentially public health interventions as species important in either amplifying or regulating the pathogen are overlooked. Investigating the evolutionary and ecological strategy of newly discovered or emerging pathogens within the community of potential and actual host species will be fruitful to both biological understanding and public health.

## 1. Introduction

Zoonotic pathogens—those transmitted among nonhuman vertebrates that can infect humans—represent an increasingly important threat to human health [1, 2]. Diseases caused by zoonotic pathogens are twice as likely as strictly human pathogens to be classified as emerging or reemerging [1]. When a zoonotic disease emerges, it is imperative that public health specialists determine the underlying cause, including identifying the pathogen and its reservoirs in nature. Identifying reservoirs, the host species that maintain the pathogen in nature, can be key to interventions to reduce disease risk but rarely are effective reservoir-based interventions realized [3]. In this essay we argue that these efforts can be substantially improved by explicit recognition that zoonotic pathogens are regulated by multiple reservoir and nonreservoir hosts that must be considered in a community context. 

In some cases, emerging zoonoses can be traced to a single-reservoir host species. For example, a recent outbreak of the Sin Nombre virus, a deadly member of the New World Hantavirus family, caused severe morbidity, mortality, and widespread panic in the Southwestern US in the early 1990s. Nearly all cases can be traced back to contact with contaminated urine or feces from the deer mouse (*Peromyscus maniculatus*), the primary reservoir species of the Sin Nombre virus. Sin Nombre virus and other related Hantavirus species tend to specialize and coevolve with a specific species of mouse [4–6].

Strict specialization of emerging zoonotic pathogens on single host species, however, appears to occur in only a minority of cases. Far more frequently, emerging zoonotic pathogens use multiple reservoir hosts. One recent example is the West Nile virus (WNV) epidemic in the United States, which has caused widespread morbidity, mortality, and concern. Unlike Hantavirus species, WNV naturally infects a tremendous array of vertebrate species including at least 300 bird species and some mammals [7–9]. In part owing to the pervasive assumption that zoonotic pathogens specialize on particular reservoir hosts, much of the public and academic discourse on WNV focuses on corvids (especially American crows) because they die conspicuously after infection and are considered harbingers of an approaching disease outbreak [8, 10–13]. This focus on corvids leads to the misperception that crows are the primary or principal reservoir of WNV [9, 14–18] when in fact they are relatively unimportant in WNV maintenance and amplification (increasing abundance in nature) due to their relative rarity at some sites and the preference of mosquitoes for other bird species such as American robins [8].

**Figure 1:**
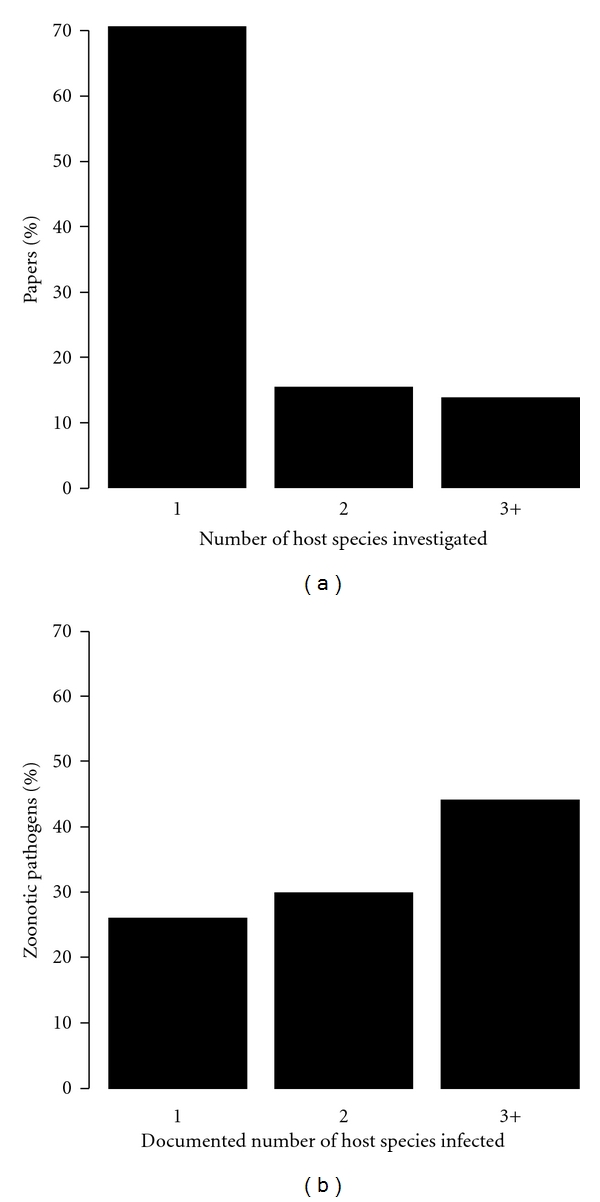
Although the vast majority of zoonotic disease scientific publications focus on a single vertebrate host species, the majority of zoonotic pathogens infect multiple host species. (a) Over 70% of the 1672 publications listed on PubMed that focus on a zoonotic pathogen investigated a single vertebrate host species. (b) By contrast, nearly 74% of the 865 zoonotic pathogens with documented host-species ranges infect multiple host species [1, 19–21].

The notion that each zoonotic pathogen has a primary or principal vertebrate reservoir species permeates the scientific literature. Over two-thirds of the publications in PubMed that investigate zoonotic pathogens and their natural reservoirs focus on only one vertebrate species (Figure 1(a)). By contrast, over two-thirds of pathogens classified as zoonoses infect multiple nonhuman vertebrate species [1, 19–21] (Figure 1(b)). Some of the studies that focus on a single host species are intended to provide specific information that can be used for later, more holistic assessments of zoonotic risk. However, in reality, the more holistic syntheses are rarely performed, preserving the paradigm that zoonotic pathogens have a primary reservoir host species. Broader syntheses are important as every infected vertebrate species in an ecological community can affect the overall reproductive success and abundance of zoonotic microbes. Further, every member of the focal community, infected or not, has the potential to affect human disease risk by contributing to the transmission dynamics and overall abundance of the pathogen in nature. Here, we advocate a more inclusive approach to understanding the underlying ecological basis of variation in zoonotic disease risk whenever time and financial constraints allow. Both the basic understanding of the causes of variable risk and the creation of effective disease management plans would benefit from explicit recognition that zoonotic pathogens are typically regulated by a community of hosts.

 Public health measures to mitigate zoonotic disease incidence often focus on preventing transmission between nonhuman vertebrates and humans or controlling infection in the nonhuman vertebrate hosts. The focus on a single reservoir species obscures the basic biological features of zoonotic pathogens. The 2003 SARS outbreak in China serves as an example. At the outset of this epidemic, epidemiologists set as a top priority the need “to identify the animal reservoir” [22]. The assumption that the SARS coronavirus (CoV) had one primary reservoir host species was so pervasive that the Chinese government mandated the culling of ~10,000 palm civets (*Paguma larvata,* see Supplemental Figure  1 in Supplementary Material available online at doi:10.1155/2011/741406) after only six individual civets from a live-animal market tested positive [23]. Subsequent investigations suggested that the infected civets likely acquired the virus in a live-animal market from another species [24]. The search for natural reservoirs of SARS-CoV has revealed just how nonspecialized this pathogen can be. Several species of horseshoe bats (*Rhinolophus* spp., Supplemental Figure  1) have been implicated as reservoirs of SARS-like CoV [23–25], but their roles in amplifying the pathogen and in transmitting it interspecifically remain obscure. Although several bat species clearly harbor active infections, with six species having >15% prevalence, none of the bats examined so far appear to transmit the virus through the respiratory tract, thought to be the primary or sole mode of transmission [25]. Active SARS-like CoV infections have been discovered in at least six nonbat species at a prevalence greater than 15%, but their roles in maintaining and transmitting SARS-like CoV similarly are unknown. Further studies are needed to quantify the roles that each host species plays in amplifying and transmitting the pathogen among species and to humans.

The distribution and abundance of multihost zoonotic pathogens—and thus disease risk—is dependent on the distribution, abundance, and behaviors of *many *animal species with which the pathogen interacts, not only the most efficient or primary reservoir species. Animals within each of these species act as the environments within which zoonotic pathogens can propagate and from which they can disperse. The total abundance of a zoonotic pathogen population is a direct function of its replication, transmission, and colonization success in each environment it colonizes, or attempts to colonize. Source-sink theory in ecology posits that organisms exist in some habitats (sinks) only because of immigration from other habitats (sources) [26]. In sink habitats, the rate of reproduction does not meet or exceed that of mortality such that sinks are not self-sustaining but instead require immigration. Sources are self-sustaining environments that can provide dispersers that might colonize sinks. Zoonotic pathogen abundance and distribution is regulated by both source and sink environments, dispersal rates between these environments, and the sizes and positions of source and sink environments. Pathogens exist in analogous landscapes consisting of multiple reservoir hosts of various quality (sources) and nonreservoirs (sinks), with the enrichment that each vertebrate species is mobile so that the landscape is constantly shifting. Hence, discovering that a species is infected with a pathogen does not necessarily mean that species is a pathogen reservoir. It could be a “sink” or spillover host the net effect of which is to *reduce* pathogen replication. Just as several habitat types can differ in their contributions to overall population growth of an animal occupant, so too can different reservoirs differ in how they contribute to the force of infection.

Below we explore the ecology of zoonotic pathogens as the complex of interactions with the animal community and relate the resulting evolutionary fitness of the pathogens to human disease risk. The evolutionary fitness, and thus the abundance, of a pathogen is dependent on a variety of factors that emerge from both the direct and indirect interactions between the pathogen and features of their environment. In this paper, we explore the effects of the direct and indirect interactions between zoonotic pathogens and (1) animal species that amplify pathogen abundance in nature; (2) animal species that negatively impact the abundance of pathogens; (3) animal species that facilitate pathogen amplification but that are not directly involved in the transmission cycle. Additionally, we explore the effects of interaction among vertebrate host species in the community on pathogen fitness. Within this framework, the evolutionary and ecological strategies of a pathogen that promote its continual existence directly affect human disease risk. Although this commentary is focused explicitly on vertebrate host species, the general argument may also be applicable to invertebrate vectors, an idea that should be explored in future work.

## 2. Ecology of Evolutionarily Successful Zoonotic Pathogens

The fitness of zoonotic pathogens is controlled by the quantity and quality of their environments, namely, each of the animal populations with which it interacts. Reservoir species are ecological sources of the pathogen in nature. These species act as “high-quality patches” that make a positive contribution to the overall abundance of the zoonotic pathogen [26, 27]. A high rate of secondary cases caused by a typical infected individual—thus increasing the abundance of the pathogen—is a hallmark of a reservoir species [28–30]. All evidence suggests that the majority of zoonotic pathogens use multiple reservoir species. We suggest that this community of reservoir species collectively maintains or increases the abundance of the zoonotic pathogen.

While species that contribute positively to the abundance of the zoonotic pathogen and to human disease risk are all qualitatively reservoirs of the pathogen, each reservoir species has a *quantitatively* different impact on pathogen abundance and human disease risk. The quantitative effect of a reservoir species on the abundance of a zoonotic pathogen is a function of five commonly used parameters [28, 31] that vary depending on the reservoir species and on the zoonotic pathogen: (1) host-species density, (2) host infection prevalence, (3) transmission rate, (4) transmission duration, and (5) the intra- and interspecific connectivity among hosts in the community. Pathogen population growth is the result of each of these species-specific parameter values integrated across all animal species in the community. Estimates of these parameters in natural settings will allow public health officials to determine the most effective set of species to target to substantially decrease disease risk, regardless of whether they are the primary reservoir species.

### 2.1. Host Density and Infection Prevalence

Host density and infection prevalence are the most commonly assessed parameters of reservoir species in nature. These parameters are rarely measured in species that are difficult to work with or are inconspicuous. For example, the power of rodents as reservoir species of the cowpox virus (*Orthopoxvirus bovis*) has been hidden behind the highly conspicuous domestic cow—the presumed “primary” reservoir species [32, 33]. Cows had been considered the primary reservoir of cowpox for at least five hundred years until urban centers (devoid of cattle) experienced a dramatic increase in human cases in the middle of the 20th century. Cows show visual signs of infection and have traditionally been associated with infected humans. In the last few decades, small rodents were identified as a natural reservoir host species [34]. New evidence suggests that the community of reservoir species is even more complex as new reservoir species continue to surface [35, 36].

### 2.2. Transmission Rate and Duration

Transmission rate and transmission duration determine the maximum number of susceptible individuals that can be infected as a result of a single infected individual [37–40]. The transmission strategies of zoonotic pathogens often depend on the species host that is infected. For example, Nipah virus infections are highly virulent in pigs and are characterized by a high level of excretion and a short transmission duration [41], while the virus in grey-headed fruit bats (*Pteropus poliocephalus*) is less virulent with low-level excretion over long time periods [42]. Virulence of a pathogen within each of its hosts has been shaped by trade-offs between the need to increase in numbers within the infected host and the need to disperse to new uninfected hosts [43]. Physiological and immunological differences between hosts will inevitably result in host-specific optima. Consequently, reservoir species often differ quantitatively in their effect on the magnitude and duration of pathogen transmission, and thus human disease risk.

### 2.3. Inter- and Intraspecific Connectivity

The evolutionary success for a zoonotic pathogen is a function of the average number of susceptible animals that become infected as a result of a single infected animal [38, 39]. The number of susceptible animals that become infected is dependent in part on the connectivity among hosts with regard to pathogen transmission. High connectivity among individuals within a reservoir species or among reservoir species contributes to increased pathogen abundance in the host community. In fact, interspecies connectivity (among reservoir species) can be essential to pathogen success. For example, cowpox cannot be maintained in a rodent community consisting solely of wood mice (*Apodemus sylvaticus*) even though wood mice are easily infected and transmit cowpox. Intraspecies connectivity among wood mice is limited such that extinction of the cowpox virus occurs shortly after introduction into a wood mouse population. However, connectivity among reservoirs is sufficient to maintain cowpox when the vertebrate community includes wood mice, bank voles (*Myodes glareolus*), and field voles (*Microtus agrestis*) [44].

## 3. Diluting Species

Zoonotic pathogens, like all species, experience environments to which they are poorly suited and in which they rarely persist. These low-quality habitats, or “dilution species,” negatively impact the abundance of pathogens and thus can decrease human disease risk [45]. Disease dilution can occur by several mechanisms, at least three of which are common in nature [46]. The first is by reducing rates of encounter between pathogens and reservoir species. For example, cattle used as zooprophylaxis to deflect malaria-inducing mosquito bites from humans reduce vector-host encounters that would allow pathogen amplification are reduced [47–49]. Similarly, the presence of predators can reduce the activity rates and home range sizes of small-mammal reservoir species, thereby reducing their encounter rates with other susceptible hosts or vectors [50, 51]. Second, the presence of species other than reservoir species can dilute disease risk by regulating abundance of the reservoir species. For example, competitors can strongly regulate the abundance of a reservoir species, reducing the overall abundance of a reservoir species and thus the pathogen [52]. Third, in vector-borne zoonoses, the presence of a variety of species can act to regulate the abundance of the vector itself, thereby modulating pathogen transmission. For example, some hosts for the tick vector of the Lyme disease bacterium, *Borrelia burgdorferi,* are poor quality hosts for ticks, killing the majority that attempt to take a bloodmeal. The result is a strong reduction in vector abundance compared with host communities in which species of low quality for ticks are rare or absent [53].

Antibody surveys are commonly used to assess disease prevalence in wildlife populations. However, serologic evidence of infection may be common in both reservoir and dilution species as both reservoir and dilution species are often exposed and may produce antibodies to the pathogen. Disease control measures aimed at species with high seroprevalence may inadvertently target a dilution species and *increase *disease risk. Many mammalian species, such as raccoons, have high seroprevalence for WNV although there is no demonstration to date that they transmit the virus to feeding vectors [54]. Mammals may reduce the prevalence of WNV by attracting mosquito bites that would have otherwise infected a reservoir species such as some passerine birds [55].

## 4. Vector Amplifiers

Vector-borne diseases comprise some of the most virulent human diseases. Plague, yellow fever, and tularemia cause severe, debilitating symptoms and are still regularly transmitted to naïve hosts due to the activity of vectors. In fact, transmission of mosquito-borne pathogens may even be enhanced by debilitating symptoms as immobilized hosts are poorer at avoiding mosquito bites than are asymptomatic hosts, resulting in longer feeding bouts that increase the probability of acquiring blood-borne pathogens [43]. Although a healthy vector population is imperative for vector-borne disease connectivity, the species that are important for maintaining the vector population often differ from those that are important for maintaining the pathogen. For example, in the far Western United States, the western fence lizard (*Sceloporus occidentalis*) is important in maintaining vector tick populations, but rodents are necessary to infect ticks with Lyme disease spirochetes [56, 57]. In such systems, controlling a nonreservoir host important in maintaining vectors might be more effective than controlling reservoir hosts that are important in transmitting the pathogen.

## 5. Community Effects

Classical ecological interactions among organisms such as competition, predation, and behavior are important regulators of zoonotic pathogen abundance and disease risk. Species that play no direct role in increasing or diluting pathogen abundance can affect pathogen abundance by regulating the density of reservoir species. For example, the density of many rodent populations is regulated by resource abundance. Recent studies show that the density, distribution, and timing of acorn production from oak trees affect Lyme disease risk by controlling the densities of white-footed mice and chipmunks—reservoir host species for Lyme disease bacteria in the Northeastern US—although oak trees themselves have no direct interaction with *B. burgdorferi *[58].

## 6. Summary and Conclusions

The majority of zoonotic pathogens infect, and are amplified by, multiple vertebrate species despite the tendency of much of the research community to focus efforts on a single “primary” reservoir species. Nonhuman vertebrate species with which a zoonotic pathogen interacts behave as environments of differing quality, such that each environment has a quantitatively different effect on pathogen prevalence and abundance and thus on disease risk. We advocate a new approach in response to newly emerging zoonotic diseases that focuses on the community of species that directly or indirectly affects the abundance of a zoonotic pathogen. This set of species is likely to include multiple reservoirs and might include dilution species as well as those that regulate reservoir or dilution species. Although targeting a set of species is more difficult than pursuing only one “primary” reservoir species, the community-based approach will help deter false leads and ineffective public health interventions that are based on an oversimplified ecology of the zoonotic pathogen. Pursuing a more inclusive set of vertebrates involved in zoonotic transmission will also be more likely to uncover cryptic yet critical interactions affecting risk.
